# Early-Onset/Young-Onset Colorectal Carcinoma: A Comparative Analysis of Morphological Features and Biomarker Profile

**DOI:** 10.7759/cureus.42340

**Published:** 2023-07-23

**Authors:** Atif A Hashmi, Mahnoor Aslam, Khushbakht Rashid, Abrahim H Ali, Tanim Ud Dowlah, Umair Arshad Malik, Shamail Zia, Sunder Sham, Fazail Zia, Muhammad Irfan

**Affiliations:** 1 Pathology, Liaquat National Hospital and Medical College, Karachi, PAK; 2 Public Health Sciences, University of Alberta, Edmonton, CAN; 3 Internal Medicine, Baqai Medical University, Karachi, PAK; 4 Internal Medicine, Liaquat National Hospital and Medical College, Karachi, PAK; 5 Internal Medicine, Bangladesh Medical College, Dhaka, BGD; 6 Internal Medicine, Aga Khan University, Karachi, PAK; 7 Pathology, Jinnah Sindh Medical University, Karachi, PAK; 8 Pathology, Lenox Hill Hospital, New York, USA; 9 Statistics, Liaquat National Hospital and Medical College, Karachi, PAK

**Keywords:** her2/neu, pms2, msh2, mlh1, immunohistochemistry, young-onset colorectal carcinoma, early-onset colorectal carcinoma, biomarkers, microsatellite instability (msi), colorectal carcinoma

## Abstract

Introduction

Colorectal carcinoma (CRC) is one of the most common cancers that involve the human body. Young-onset CRC (YO-CRC) or early-onset CRC (EO-CRC) is defined as CRC that develops before the age of 50 years, as opposed to CRC that is diagnosed after the age of 50, referred to as late-onset CRC (LO-CRC). EO-CRC is sparsely studied in our population. Therefore, in this study, we evaluated the clinicopathological parameters and biomarker profile of EO-CRC and compared them with those of LO-CRC.

Methods

This was a retrospective study conducted at the Department of Histopathology, Liaquat National Hospital, Karachi, Pakistan. A total of 254 biopsy-proven cases of CRC, reported over a period of nine years, were enrolled in the study. The specimens collected during surgery were sent to the laboratory for histopathological and immunohistochemical (IHC) status examinations. IHC staining of the specimens was performed using antibodies, namely, MutL protein homolog 1 (MLH1), postmeiotic segregation increased 2 (PMS2), MutS homolog 2 (MSH2), MutS homolog 6 (MSH6), and human epidermal growth factor receptor 2 (HER2/neu), on representative tissue blocks. A comparison of morphological and biomarker profiles between EO-CRC and LO-CRC was performed.

Results

The mean age at diagnosis was 46.27±17.75 years, with female predominance (59.8%). A significant difference between the two groups (EO-CRC and LO-CRC) was noted with respect to laterality, tumor site, tumor grade, tumor type, presence of pre-existing polyps, perineural invasion (PNI), lymphovascular invasion (LVI), and IHC markers. EO-CRC (as opposed to LO-CRC) significantly affected the left colon (92.6% vs. 72.9%, p<0.001), with the rectosigmoid being the most common site in the majority of cases (72.1% in EO-CRC vs. 61% in LO-CRC). EO-CRC showed a higher frequency of PNI and LVI than LO-CRC (42.6% vs. 23.7%, p=0.001; 29.4% vs. 18.6%, p=0.046, respectively). A significantly higher proportion of EO-CRCs were mucinous (42.6%) and medullary carcinoma (11.8%). Although the majority (54.4%) of cases of EO-CRC were grade 2 tumors at the time of diagnosis, a significantly higher proportion of them were grade 3 (44.1%) compared with LO-CRC. IHC comparisons between the two age groups showed that a significantly higher proportion of cases of EO-CRC showed positive HER2/neu expression (27.1%) compared with LO-CRC (13.2%). Conversely, the loss of expression of microsatellite instability (MSI) markers was more commonly seen in LO-CRS compared with EO-CRC.

Conclusions

We found a relatively higher frequency of EO-CRC in our population. Moreover, compared with LO-CRCs, EO-CRCs were associated with prognostically poor histological parameters, such as mucinous and medullary carcinoma, high-grade, PNI, and LVI. Similarly, EO-CRC had a higher positive expression of HER2/neu with intact MSI markers compared with AO-CRC; all these characteristics indicate poor biological behavior in EO-CRC.

## Introduction

Colorectal carcinoma (CRC) is one of the leading cancers worldwide, with 1.9 million new cases and 935,000 deaths estimated to be reported in 2020, making it the third most common cancer globally and the second most common cancer in terms of mortality [1]. Over the past few decades, it has been observed that the overall incidence of CRC in the United States of America has declined at a rate of approximately 2-3% per year in both genders, whereas it has also been observed that the rate of CRC in younger individuals, below 50 years of age, has increased [2,3]. The decline in the rate of CRC occurring after 50 years of age, also termed late-onset CRC (LO-CRC), is attributed to the adoption of a healthy lifestyle, screening colonoscopy (enabling early detection), removal of premalignant lesions, and advancement in treatment [2].

CRC not meeting the usual age criteria and occurring in younger people of age below 50 years is termed "early-onset CRC" (EO-CRC) or "young-onset CRC" (YO-CRC) [4]. The reason behind the upward trend in the incidence of EO-CRC is still unclear, but it may be attributed to certain modifiable risk factors, for instance, the prevalence of a sedentary lifestyle, obesity, change in dietary habits (such as consumption of alcohol and processed food), and adoption of a westernized lifestyle and non-modifiable risk factors (e.g. race, ethnicity, and heredity) [5,6].

Three main carcinogenic pathways are involved in the development of CRC, namely, chromosomal instability (CIN), microsatellite instability (MSI), and aberrant methylation of CpG islands (CIMP) [3]. It has been reported that CIN is the most common pathway by which most CRC develops, the majority of which are sporadic; and 12-15% develop via the MSI pathway, which is the result of deficient DNA mismatch repair (dMMR) [7]. dMMR can be attributed to an inherited germline mutation in the mismatch repair (MMR) genes, commonly known as Lynch syndrome, constituting one-third of MSI tumor cases, with two-thirds being sporadic [7]. Certain biomarkers of CRC carcinogenesis are considered to have significant prognostic and predictive value, MSI being one of them. In 2014, the National Comprehensive Cancer Network (NCCN) recommended that MSI be tested universally in all cases of CRC [8].

Microsatellites are composed of tandem repeats of one to six nucleotides, located in the coding and non-coding parts of the genome [9]. During DNA replication, the MMR system ensures the stability of the microsatellites, and failure of this system results in a high level of MSI (MSI-H) [10].

Approximately 2-3% of CRCs manifest human epidermal growth factor 2 (HER2/neu) overexpression. Several reports have concluded that screening of HER2/neu has significant prognostic and therapeutic value and has consequently been identified as an important biomarker for CRC [11].

Scarce and conflicting data are available on the site, histology, tumor stage, and biomarker profile of EO-CRC. Therefore, in this study, we performed histological and biomarker evaluations of CRC and compared the findings of EO-CRC with LO-CRC. Immunohistochemical (IHC) studies were used to determine the biomarker profile of CRC. The study outlined the distinguishing morphological features and biomarker profile of EO-CRC, comparing it with LO-CRC for a better understanding of the pathogenesis of EO-CRC. Currently, both EO-CRC and LO-CRC are treated similarly; a better understanding of the disease could provide us with an opportunity to devise screening and management strategies that improve the overall outcome of the disease.

## Materials and methods

This is a retrospective, cross-sectional study conducted at the Department of Histopathology, Liaquat National Hospital, Karachi, Pakistan. A total of 254 cases of CRC reported at our institute over the period of nine years were enrolled in the study. Data on CRC cases reported between January 2012 and December 2020 were retrieved from the institute archives. All the cases included were biopsy-proven CRC cases, and all the patients underwent surgical resection of the primary tumor. Cases with incomplete clinical or surgical records were excluded from the study. Cases that received neoadjuvant chemotherapy before surgical resection were also excluded from the study. All the tissue specimens obtained during the surgery were sent to the laboratory for histopathology and IHC staining. After the gross examination, the samples were prepared for histological and IHC evaluation.

All resection specimens were received in the histopathology lab. Followed by overnight fixation, specimens were grossly examined for the location, size, color, texture, and appearance of the tumor and its distance from the resection margins. Representative sections were then submitted from the tumor (one section per cm), margins, normal appearing mucosa, any abnormal mucosa, and lymph nodes.

For histological examination, the specimens obtained were fixed with 10% neutralized formalin, which was then washed with distilled water, followed by being treated with increasing concentrations of alcohol (70%, 90%, 96%, and 100%), incubated for three hours in xylene, and finally embedded in paraffin at 56°C. The paraffin blocks were sliced into 4-um-thick sections, which were then placed on slides treated with lysine, which were dried and subsequently stained with hematoxylin and eosin and were studied under a microscope. Various morphological features were noted. Tumor typing and grading were performed as per World Health Organization (WHO) guidelines. Tumor-node-metastasis (TNM) staging was performed according to the American Joint Committee on Cancer (AJCC) guidelines.

IHC staining

The IHC staining for MSI proteins was performed on a four-antibody panel of MMR proteins, namely, MutL protein homolog 1 (MLH1), postmeiotic segregation increased 2 (PMS2), MutS homolog 2 (MSH2), and MutS homolog 6 (MSH6). Staining was also performed for HER2/neu on representative paraffin-fixed tissue blocks. From the unstained paraffin blocks, 4-um-thick sections were sliced and deparaffinized in xylene, and rehydrated in alcohol, and then the samples were washed in distilled water. For antigen retrieval, the paraffin-embedded sections were pretreated at 97°C with heat-induced epitope retrieval (HIER) for 35-40 minutes at a high pH (50 x). The antibodies were in ready-to-use form in a buffer containing staining protein and 0.015 mol/L sodium azide (MSH6 clone, EP49; MSH2 clone, FE11; PMS2 clone, EP51; MLH1 clone, ES05). The slides prepared were incubated with the following antibodies: MLH1, MSH2, MSH6, and PMS2.

IHC staining was done manually in batches of 10, with positive and negative controls running along with each batch. IHC interpretation was done according to the American Society of Clinical Oncology (ASCO)/College of American Pathologists (CAP) protocol: any (even patchy) nuclear staining in tumor cells was taken as "no loss of expression," while tumor cells with absolutely no staining were marked as "loss of expression," presuming that internal controls were positive [7]. Normal adjacent colonic epithelium, lymphocytes, and stromal cells were used as positive internal controls. IHC expression of proteins was categorized into five groups: no loss of expression, the loss of expression of all four proteins, isolated loss of MLH1, combined loss of MLH1/PMS2, and combined loss of MSH2/MSH6. Hence, CRC with any absence of nuclear staining (even patchy or focal) for at least one protein was considered dMMR.

IHC scoring of HER2/neu was performed according to the CAP guidelines for HER2/neu breast cancer (on a scale of 0/negative to 3+/positive), and evaluation of only membranous HER2/neu expression was performed. Two factors were taken into account, namely, the intensity of expression and the percentage of positively stained cells [12]. Furthermore, molecular testing of cases with equivocal (2+) IHC expression was done by the fluorescent in situ hybridization (FISH) technique. The Food and Drug Administration (FDA)-approved Path Vysion HER2-DNA probe kit was used for FISH testing. The interpretation of results was specified according to the ASCO/CAP recommendation as negative (not amplified) or positive (amplified) [12].

Data analysis

Data analysis was performed using the Statistical Package for Social Science (SPSS, Version 26.0; IBM Inc., Armonk, NY, USA). The mean and standard deviation were calculated for the age, whereas frequencies and percentages were evaluated for all other clinicopathological variables. A p-value of <0.05 was considered significant. Chi-square and Fisher’s exact tests were applied to determine the association of various clinicopathological features with respect to age groups.

## Results

A total of 254 cases of CRC were included in the study. Table 1 demonstrates the clinicopathological profile of the cases included in the study. The mean age at the time of diagnosis was found to be 46.2±17.75 years. In our study, CRCs were more prevalent in younger patients of less than 50 years of age, accounting for 53.5% of cases, while 46.5% of the patients were diagnosed at an older age of more than 50 years. Anatomically, CRCs were more common on the left side (83.5%), whereas right-sided CRCs were diagnosed in only 16.5% of cases. Recto-sigmoid was the most common site, occurring in 66.9% of cases, followed by splenic flexure (12.6%) and cecum (11%). Histological examination showed that CRCs showed perineural invasion (PNI) in 33.9% of cases, lymphovascular invasion (LVI) was present in 24.4% of cases, and pre-existing polyps were seen in only 6.3% of cases. The most common tumor type was found to be adenocarcinoma, no special type (NOS), identified in 63.8% of cases; the second most common type diagnosed was mucinous adenocarcinoma (26%), followed by medullary carcinoma (7.1%) and signet cell adenocarcinoma (3.1%). The majority (67.7%) of cases were found to be grade 2 tumors (moderately differentiated). TNM staging of the tumors demonstrated that the majority (71.6%) of cases were diagnosed at a later stage at the tumor (T) stage T3, whereas 19.7% of cases at the time of diagnosis were at stage T4. Nodal (N) metastasis was found to be present in 66.9% of cases at the time of diagnosis, and perinodal extension was seen to be present in 52.8% of cases. Host immune responses (i.e. peritumoral lymphocytic infiltration (PTL) and intratumoral lymphocytic infiltration (ITL)) were observed and represented in Table 1. IHC staining of the tumors showed that 19.7% of the tumors were HER2/neu-positive. Among the MMR proteins, loss of expression for MLH1 was seen in 38.6% of cases; loss of expression for PMS2 was seen in 33.9% of cases; MSH2 loss of expression was seen in 18.9% of cases; and the loss of expression for MSH6 was seen in 17.3% of cases. Out of 254 cases, 152 cases (59.8%) showed intact expression of all markers, 36 cases (14.2%) showed a loss of expression of all markers, 46 cases (18.1%) showed a combined loss of expression of MLH1 and PMS2, eight cases (3.1%) showed a combined loss of MSH2 and MSH6, and 12 cases (4.7%) showed an isolated loss of MLH1, as shown in Table 1.

**Table 1:** Clinicopathological features of the population under study SD: standard deviation; NOS: not otherwise specified; T: tumor; N: nodal; HER2/neu: human epidermal growth factor receptor 2; MLH1: MutL protein homolog 1; PMS2: postmeiotic segregation increased 2; MSH2: MutS homolog 2; MSH6: MutS homolog 6; MSI: microsatellite instability

Clinicopathological features	Values
Age (years)	
Mean± SD	46.27±17.75
Age groups	
≤50 years, n (%)	136 (53.5)
>50 years, n (%)	118 (46.5)
Gender	
Male, n (%)	102 (40.2)
Female, n (%)	152 (59.8)
Laterality	
Right, n (%)	42 (16.5)
Left, n (%)	212 (83.5)
Tumor location	
Cecum, n (%)	28 (11)
Ascending colon, n (%)	10 (3.9)
Transverse colon, n (%)	12(4.7)
Recto sigmoid, n (%)	170 (66.9)
Descending colon, n (%)	2 (0.8)
Splenic flexure, n (%)	32 (12.6)
Histological features	
Perineural invasion	
Present, n (%)	86 (33.9)
Absent, n (%)	168 (66.1)
Lymphovascular invasion	
Present, n (%)	62 (24.4)
Absent, n (%)	192 (75.6)
Pre-existing polyp	
Present, n (%)	16 (6.3)
Absent, n (%)	238 (93.7)
Tumor type	
Adenocarcinoma, NOS, n (%)	162 (63.8)
Mucinous adenocarcinoma, n (%)	66 (26)
Medullary carcinoma, n (%)	18 (7.1)
Signet ring cell adenocarcinoma, n (%)	8 (3.1)
Tumor differentiation/grade	
Well-differentiated/ grade 1, n (%)	8 (3.1)
Moderately differentiated/ grade 2, n (%)	172 (67.7)
Poorly differentiated/ grade 3, n (%)	74 (29.1)
Tumor (T)-stage	
T1, n (%)	2 (0.8)
T2, n (%)	20 (7.9)
T3, n (%)	182 (71.6)
T4, n (%)	50 (19.7)
Nodal metastasis	
Present, n (%)	170 (66.9)
Absent, n (%)	84 (33.1)
Nodal (N)-stage	
N0, n (%)	84 (33.1)
N1, n (%)	56 (22)
N2a, n (%)	64 (25.2)
N2b, n (%)	50 (19.7)
Perinodal extension	
Present, n (%)	134 (52.8)
Absent, n (%)	120 (47.2)
Signet ring cell differentiation	
Present, n (%)	46 (18.1)
Absent, n (%)	208 (81.9)
Mucinous differentiation	
Present, n (%)	96 (37.8)
Absent, n (%)	158 (62.2)
Cribriform pattern	
Present, n (%)	46 (18.1)
Absent, n (%)	208 (81.9)
Necrosis	
Absent, n (%)	180 (70.9)
Present, focal, n (%)	46 (18.1)
Present, widespread, n (%)	28 (11)
Peritumoral lymphocytic infiltrate	
None, n (%)	202 (79.5)
Mild to moderate, n (%)	28 (11)
Marked, n (%)	24 (9.4)
Intratumoral lymphocytic infiltrate	
None, n (%)	142 (55.9)
Mild to moderate, n (%)	72 (28.3)
Marked, n (%)	40 (15.7)
Biomarker/ immunohistochemical profile	
HER2/neu	
Positive, n (%)	50 (19.7)
Negative, n (%)	204 (80.3)
MLH1	
Intact nuclear expression, n (%)	156 (61.4)
Loss of expression, n (%)	98 (38.6)
PMS2	
Intact nuclear expression, n (%)	168 (66.1)
Loss of expression, n (%)	86 (33.9)
MSH2	
Intact nuclear expression, n (%)	206 (81.1)
Loss of expression, n (%)	48 (18.9)
MSH6	
Intact nuclear expression, n (%)	210 (82.7)
Loss of expression, n (%)	44 (17.3)
Overall MSI status	
Intact expression of all markers, n (%)	152 (59.8)
Loss of expression of all markers, n (%)	36 (14.2)
Loss of MLH and PMS2, n (%)	46 (18.1)
Loss of MSH2 and MSH6, n (%)	8 (3.1)
Isolated loss of MLH1 only, n (%)	12 (4.7)

Table 2 depicts the comparison of the clinicopathological features of EO-CRC with LO-CRC. A significant difference between the two groups was noted with respect to laterality, tumor site, tumor grade, tumor type, the presence of pre-existing polyps, PNI, LVI, and IHC markers. Although both EO-CRC and LO-CRC were more common on the left side, a higher proportion of left-sided tumors were EO-CRC. Similarly, in both age groups, the most common site was recto-sigmoid; however, a significantly higher proportion of splenic flexure tumors were EO-CRC. Comparison of histological features between the two age groups showed that PNI and LVI were significantly more likely to be associated with EO-CRC (42.6% and 29.4%, respectively), compared with LO-CRC (23.7% and 18.6%, respectively). Similarly, a significantly higher proportion of EO-CRCs were mucinous (42.6%) and medullary carcinoma (11.8%). Although the majority (54.4%) of cases of EO-CRC were grade 2 tumors at the time of diagnosis, a significantly higher proportion of them were grade 3 (44.1%) compared with LO-CRC, among which 11.9% were grade 3. The TNM staging comparison between the two age groups did not show any significant difference, except that the N2b stage was more frequently seen in EO-CRC. The majority (72.1%) of EO-CRC cases were found to be at the T3 stage, followed by 22.1% at the T4 stage. Similarly, in the LO-CRC, the majority (71.2%) of cases were at the T3 stage, followed by 16.9% at the T4 stage, and 11.9% at the T2 stage. Nodal metastasis in EO-CRC was seen in 64.7% of cases, whereas, in LO-CRC, the percentage of nodal metastasis was slightly higher; it was found to be present in 69.5% of cases; however, the difference is not statistically significant. Pre-existing polyps were more likely to be associated with LO-CRC, occurring in 10.2% of cases compared with EO-CRC (2.9%). A higher proportion (48.5%) of EO-CRC showed mucinous features compared with LO-CRC (25.4%). Conversely, cribriforming was more commonly seen in LO-CRC. The evaluation of two host immune responses (i.e. PTL and ITL) showed that EO-CRC showed a higher frequency of marked PTL (10.3% in EO-CRC vs. 8.3% in LO-CRC), while LO-CRC showed a slightly higher frequency ITL (16.9% in LO-CRC vs. 14.7% in EO-CRC). IHC comparison between the two age groups showed that a significantly higher proportion of cases of EO-CRC showed positive HER2/neu expression (27.1%) compared with LO-CRC (13.2%). Conversely, the loss of expression of MSI markers was more commonly seen in LO-CRS compared with EO-CRC (Table 2).

**Table 2:** Comparison of the clinicopathological features and biomarker profile of early-onset colorectal carcinoma with late-onset colorectal carcinoma *Chi-square test was applied, **Fisher’s exact test was applied, ***p-value significant as < 0.05 NOS: not otherwise specified; T: tumor; N: nodal; HER2/neu: human epidermal growth factor receptor 2; MLH1: MutL protein homolog 1; PMS2: postmeiotic segregation increased 2; MSH2: MutS homolog 2; MSH6: MutS homolog 6; MSI: microsatellite instability

Clinicopathological features	Values		p-value
	Early-onset colorectal carcinoma	Late-onset colorectal carcinoma	
Gender*			
Male, n (%)	60 (44.1)	42 (35.6)	0.167
Female, n (%)	76 (55.9)	76 (64.4)	
Laterality*			
Right, n (%)	10 (7.4)	32 (27.1)	<0.001***
Left, n (%)	126 (92.6)	86 (72.9)	
Location**			
Cecum, n (%)	6 (4.4)	22 (18.6)	<0.001***
Ascending colon, n (%)	2 (1.5)	8 (6.8)	
Transverse colon, n (%)	2 (1.5)	10 (8.5)	
Recto sigmoid, n (%)	98 (72.1)	72 (61)	
Descending colon, n (%)	2 (1.5)	0 (0)	
Splenic flexure, n (%)	26 (19.1)	6 (5.1)	
Histopathological features			
Perineural invasion*			
Present, n (%)	58 (42.6)	28 (23.7)	0.001***
Absent, n (%)	78 (57.4)	90 (76.3)	
Lymphovascular invasion*			
Present, n (%)	40 (29.4)	22 (18.6)	0.046***
Absent, n (%)	96 (70.6)	96 (81.4)	
Pre-existing polyp*			
Present, n (%)	4 (2.9)	12 (10.2)	0.018***
Absent, n (%)	132 (97.1)	106 (89.8)	
Tumor type*			
Adenocarcinoma NOS, n (%)	58 (42.6)	104 (88.1)	<0.001***
Mucinous adenocarcinoma, n (%)	58 (42.6)	8 (6.8)	
Medullary carcinoma, n (%)	16 (11.8)	2 (1.7)	
Signet ring cell adenocarcinoma, n (%)	4 (2.9)	4 (3.4)	
Tumor differentiation/ grade**			
Well differentiated/ grade 1, n (%)	2 (1.5)	6 (5.1)	<0.001***
Moderately differentiated/ grade 2, n (%)	74 (54.4)	98 (83.1)	
Poorly differentiated/ grade 3, n (%)	60 (44.1)	14 (11.9)	
Tumor (T)-stage**			
T1, n (%)	2 (1.5)	0 (0)	
T2, n (%)	6 (4.4)	14 (11.9)	0.062
T3, n (%)	99 (72.1)	84 (71.2)	
T4, n (%)	30 (22.1)	20 (16.9)	
Nodal metastasis*			
Present, n (%)	88 (64.7)	82 (69.5)	0.419
Absent, n (%)	48 (35.3)	36 (30.5)	
Nodal (N)-stage*			
N0, n (%)	48 (35.3)	36 (30.5)	<0.001***
N1, n (%)	20 (14.7)	36 (30.5)	
N2a, n (%)	28 (20.6)	36 (30.5)	
N2b, n (%)	40 (29.4)	10 (8.5)	
Perinodal extension*			
Present, n (%)	72 (52.9)	62 (52.5)	0.949
Absent, n (%)	64 (47.1)	56 (47.5)	
Signet ring cell differentiation*			
Present, n (%)	36 (26.5)	10 (8.5)	<0.001***
Absent, n (%)	100 (73.5)	108 (91.5)	
Mucinous differentiation*			
Present, n (%)	66 (48.5)	30 (25.4)	<0.001***
Absent, n (%)	70 (51.5)	88 (74.6)	
Cribriform pattern*			
Present, n (%)	16 (11.8)	30 (25.4)	0.005***
Absent, n (%)	120 (88.2)	88 (74.6)	
Necrosis*			
Absent, n (%)	104 (76.5)	76 (64.4)	0.002***
Present, focal, n (%)	14 (10.3)	32 (27.1)	
Present, widespread, n (%)	18 (13.2)	10 (8.5)	
Peritumoral lymphocytic infiltrate*			
None, n (%)	116 (85.3)	86 (72.9)	0.001***
Mild to moderate, n (%)	6 (4.4)	22 (18.6)	
Marked, n (%)	14 (10.3)	10 (8.5)	
Intratumoral lymphocytic infiltrate*			
None, n (%)	88 (64.7)	54 (45.8)	0.005***
Mild to moderate, n (%)	28 (20.6)	44 (37.3)	
Marked, n (%)	20 (14.7)	20 (16.9)	
Biomarker/ immunohistochemical profile			
HER2/neu*			
Positive, n (%)	32 (27.1)	18 (13.2)	0.006***
Negative, n (%)	86 (72.9)	118 (86.8)	
MLH1*			
Intact nuclear expression, n (%)	96 (70.6)	60 (50.8)	0.001***
Loss of expression, n (%)	40 (29.4)	58 (49.2)	
PMS2*			
Intact nuclear expression, n (%)	96 (70.6)	72 (61)	0.108
Loss of expression, n (%)	40 (29.4)	46 (39)	
MSH2*			
Intact nuclear expression, n (%)	112 (82.4)	94 (79.7)	0.585
Loss of expression, n (%)	24 (17.6)	24 (20.3)	
MSH6*			
Intact nuclear expression, n (%)	116 (85.3)	94 (79.7)	0.237
Loss of expression, n (%)	20 (14.7)	24 (20.3)	
Overall MSI status**			
Intact expression of all markers, n (%)	98 (72.1)	54 (45.8)	<0.001***
Loss of expression of all markers, n (%)	18 (13.2)	18 (15.3)	
Loss of MLH and PMS2, n (%)	18 (13.2)	28 (23.7)	
Loss of MSH2 and MSH6, n (%)	2 (1.5)	6 (5.1)	
Isolated loss of MLH1 only, n (%)	0 (0)	12 (10.2)	

## Discussion

The study was conducted to compare the morphological and biomarker profiles of EO-CRC with those of LO-CRC. We found a relatively high frequency of EO-CRC in our setup. Moreover, we noted a relatively higher frequency of loss of MSI markers in our cohort of cases of CRC, whereas HER2/neu expression was relatively low. Compared to LO-CRC, EO-CRC showed a higher frequency of HER2/neu expression and a lower frequency of dMMR. Poor histological parameters, such as poor differentiation, mucinous features, and PNI, were also associated with EO-CRC in our study.

Similar to our study, various studies have found that mucinous and signet ring cell features and poorly differentiated histology were more prevalent in EO-CRC compared with LO-CR; all of these characteristics are associated with an adverse prognosis [6,13]. A study by Mauri et al. found that EO-CRC were more likely to be diagnosed at an advanced stage compared with LO-CRC, with poor differentiation, increased prevalence of mucinous, and signet ring cell features and were found to more commonly occur on the left-sided colon (mostly affecting the distal colon and rectum) [3].

LVI, PNI, poor differentiation, and signet ring cell histology are pathological characteristics correlated with the presentation of CRC at an advanced stage and an adverse prognosis [14]. Our study suggested that these characteristics were more prevalent in EO-CRC compared with LO-CRC. Similar to our findings, a study by Kibriya et al. also found that PNI and LVI were significantly more frequently present in EO-CRC than in LO-CRC [15]. Yantiss et al., in another study, also concluded that EO-CRC was more frequently associated with LVI (81%) and venous invasion (48%) [16]. A study by Myers et al. confirmed that EO-CRC most commonly occurred distal to the splenic flexure (77%) and was most likely to present at an advanced stage (56% stage 3 or 4), compared with LO-CRC [17]. Moreover, features such as pre-existing polyps and positive ITL were correlated with both the lower T and N stages [18]. Our study showed that pre-existing polyps and marked ITL were more likely to be associated with LO-CRC than EO-CRC, while the frequency of marked PTL was higher in EO-CRC. 

The presence of MSI in CRC is of eminent clinical importance and has promising prognostic value [19]. MSI-H tumors have an improved prognosis compared with tumors with low microsatellite instability (MSI-L) or tumors that are microsatellite stable (MSS) [20]. Although the gold standard for MSI testing is molecular evaluation, IHC analysis is considered a surrogate for molecular evaluation. EO-CRCs or YO-CRCs, in the majority of cases, are MSS and do not possess abnormalities in the DNA repair mechanism [21]. Antelo et al. found the frequency of dMMR in cases of EO-CRC to be approximately 20%. EO-CRC with MSI-H is mostly related to inherited etiologies (Lynch syndrome) and is rarely present in sporadic cases of YO-CRC [22].

We found that the majority (72.1%) of cases of EO-CRC showed intact expression for MSI markers, whereas 13.2% of cases showed loss of expression of all markers, and 13.2% revealed combined loss of expression for MLH1 and PMS2. Conversely, LO-CRC showed a significant association with dMMR proteins, with 15.3% of cases showing loss of expression of all markers and 23.7% showing combined loss of expression of MLH1 and PMS2. Our finding that most of the cases of EO-CRC are MSS is consistent with several studies. These studies also concluded that EO-CRCs were less likely to show MSI [16,23,24]. Contrarily, some studies corroborate the finding that EO-CRC were more likely to show dMMR markers [3,25]. It has been reported that tumors that show MSI-H have a significant association with a prominent immune cell response within the tumor microenvironment (TME) [26,27]. In a study by Richards et al., they found that tumors that lacked an immune host response, such as ITL or PTL, had significantly worse prognoses than tumors that showed either of those [28].

HER2/neu expression in CRC has correlated significantly with higher tumor grade and positive nodal status and is associated with a poor prognosis [29]. Our study showed that EO-CRC expressed positive HER2/neu expression in a higher proportion of cases (27.1%), compared with LO-CRC, which expressed HER2/neu positivity in 13.2% of cases.

Limitations of the study

Our study had a few limitations, such as a limited sample size as this was a single-center study. Moreover, as this was a retrospective study, follow-up of patients was not determined, and evaluation and stratification of risk factors and molecular evaluation (molecular studies for BRAF, MLH1 promotor hypermethylation, and germline mutation analysis for MSI to evaluate Lynch syndrome) of the tumors were not performed. Therefore, we propose that further prospective multi-center studies be conducted to better understand the clinical, pathological, and histological differences between EO-CRC and LO-CRC.

## Conclusions

We found a relatively high frequency of EO-CRC in our setup. Moreover, EO-CRC was found to be associated with poor histological parameters, such as a higher histological grade or poor differentiation, a higher frequency of mucinous and medullary differentiation, the presence of PNI and LVI, and a higher nodal stage, compared to LO-CRC. Similarly, EO-CRC was associated with a higher frequency of HER/neu positivity and intact expression of MSI markers. Both of these features are associated with a poor prognosis, signifying the comparatively poor biological behavior of EO-CRC. Overall, in our studied cases, the frequency of dMMR was relatively high, while HER2/neu expression was low. As molecular studies, risk factor evaluation, and clinical follow-up were not performed in our study, we suggest future studies cover these features of EO-CRC in our population.
